# Circular RNA hsa_circ_0067842 facilitates tumor metastasis and immune escape in breast cancer through HuR/CMTM6/PD-L1 axis

**DOI:** 10.1186/s13062-023-00397-3

**Published:** 2023-08-18

**Authors:** Juan Li, Xiangjun Dong, Xue Kong, Yafen Wang, Yanru Li, Yao Tong, Wenjing Zhao, Weili Duan, Peilong Li, Yanqun Wang, Chuanxin Wang

**Affiliations:** 1 Department of Clinical Laboratory, The Second Hospital of Shandong University, Jinan, 250033 Shandong China; 2 Pathology Tissue Bank, Qilu Hospital of Shandong University, Jinan, 250012 Shandong China; 3 Department of Clinical Laboratory, The 960th Hospital of the PLA Joint Logistics Support Force, Jinan, 250031 Shandong China

**Keywords:** Breast cancer, Circular RNA, HuR, PD-L1, Metastasis, Immune escape

## Abstract

**Background:**

Circular RNAs (circRNAs) have been shown to play diverse biological functions in the progression of multiple diseases. However, the impacts of circRNAs on breast cancer (BC) progression remains unclear. Therefore, the objective of this paper is to investigate the role and mechanisms of a functional circRNA in BC metastasis and immune escape.

**Methods:**

This study used a circRNA microarray and identified a novel circRNA hsa_circ_0067842. The validation and characteristics of hsa_circ_0067842 were investigated using qRT-PCR, sanger sequencing, RNase R treatment, actinomycin D treatment and fluorescence in situ hybridization (FISH). Gain- and loss-of-function assays were performed to evaluate the biological function of hsa_circ_0067842 in BC progression and immune escape. Mechanistically, the interaction between hsa_circ_0067842 and HuR was explored by RNA pull down, mass spectrometry (MS), subcellular component protein extraction and immunofluorescence (IF). The regulatory mechanisms of hsa_circ_0067842/HuR/CMTM6/PD-L1 axis were investigated by qRT-PCR, western blot, FISH, immunoprecipitation and rescue assays.

**Results:**

The expression of hsa_circ_0067842 was upregulated in BC tissues and cells, which was found to be significantly associated with poor prognosis, regardless of other clinical covariates. Function assays showed that hsa_circ_0067842 promoted the migration and invasion capacities of BC cells. Moreover, co-culture experiment with peripheral blood mononuclear cells (PBMCs) showed that hsa_circ_0067842 played a role in the immune escape of BC cells. Mechanistically, our study showed that hsa_circ_0067842 interacted with HuR, affecting its nuclear translocation, thus enhancing the stability of CMTM6. CMTM6 not only enhances the migration and invasion ability of BC cells, but also affects the ubiquitination of PD-L1 and inhibits its degradation.

**Conclusion:**

Collectively, our results demonstrated that hsa_circ_0067842 promoted BC progression through the HuR/CMTM6/PD-L1 axis, providing new insight and a potential target for BC prognosis and therapy.

**Supplementary Information:**

The online version contains supplementary material available at 10.1186/s13062-023-00397-3.

## Introduction

BC is a global health problem and the most common cancer type among women worldwide. It has the second highest incidence and the highest mortality among women [1]. Despite advancements in diagnostic and therapeutic techniques, patients with advanced metastasis still have poor survival. Historically, BC is considered as a non-immunogenic disease, but recent investigations have shown that it can elicit host antitumor immune responses, and immunotherapy is beneficial for a subset of BC patients, particularly those with metastatic BC [2]. The programmed death ligand-1 (PD-L1), known as one of the most important immune checkpoints, is believed to have a significant impact on BC treatment. It works by inhibiting immune cell function, such as T lymphocytes, through binding to programmed death-1 (PD-1) receptor on the surface of activated immune cells, thereby helping tumor cells evade host immune surveillance [3–5]. Previous studies have revealed that BC with high PD-L1 expression is more aggressive and leads to shorter survival time [6]. Thus, understanding the mechanism of BC metastasis and immune escape is crucial to improving the prognosis and quality of life of BC patients, making it an urgent matter for researchers.

CircRNAs are a unique type of noncoding RNAs characterized by a covalently closed loop structure without a 5’-end cap and 3’-end poly A tail, making them more stable than liner RNAs and resistant to RNA exonucleases. Despite being discovered in 1976, the significance of circRNAs has only recently come to the forefront [7–9]. In BC cancer, circRNAs can act as oncogenes or suppressors in various biological processes such as cell proliferation, apoptosis, etc. For example, circ-CSNK1G1 was found to enhance the proliferation, migration, invasion and glycolysis metabolism of triple-negative breast cancer (TNBC) cell through the miR-28-5p/LDHA pathway [10]. Yang et al. identified a novel circRNA interacted with USP10, thus promoting BC progression by destabilizing p53 [11]. Furthermore, the development of high-throughput sequencing has led to the discovery of many new circRNAs with unknown biological functions and clinical potentials. However, their mechanisms and clinical value in BC progression, especially in the regulation of PD-L1, remain to be investigated.

In this study, we showed that a novel circRNA, hsa_circ_0067842, which was found to be upregulated in BC tissues, was significantly associated with poor prognosis. Our findings also showed that hsa_circ_0067842 could promote BC metastasis and immune escape. Mechanically, we discovered that hsa_circ_0067842 facilitated the translocation of HuR into the cytoplasm through its interaction with HuR. Cytoplasmic HuR then enhanced the stability of CMTM6, thus directly accelerating BC metastasis. At the same time, cytoplasmic HuR regulated the ubiquitination of PD-L1 via CMTM6, thereby affecting immune escape. In summary, our data showed that hsa_circ_0067842 was a pivotal regulator of BC progression and might serve as a potential therapeutic target.

## Results

### Identification and characterization of hsa_circ_0067842 in BC

**Fig. 1:**
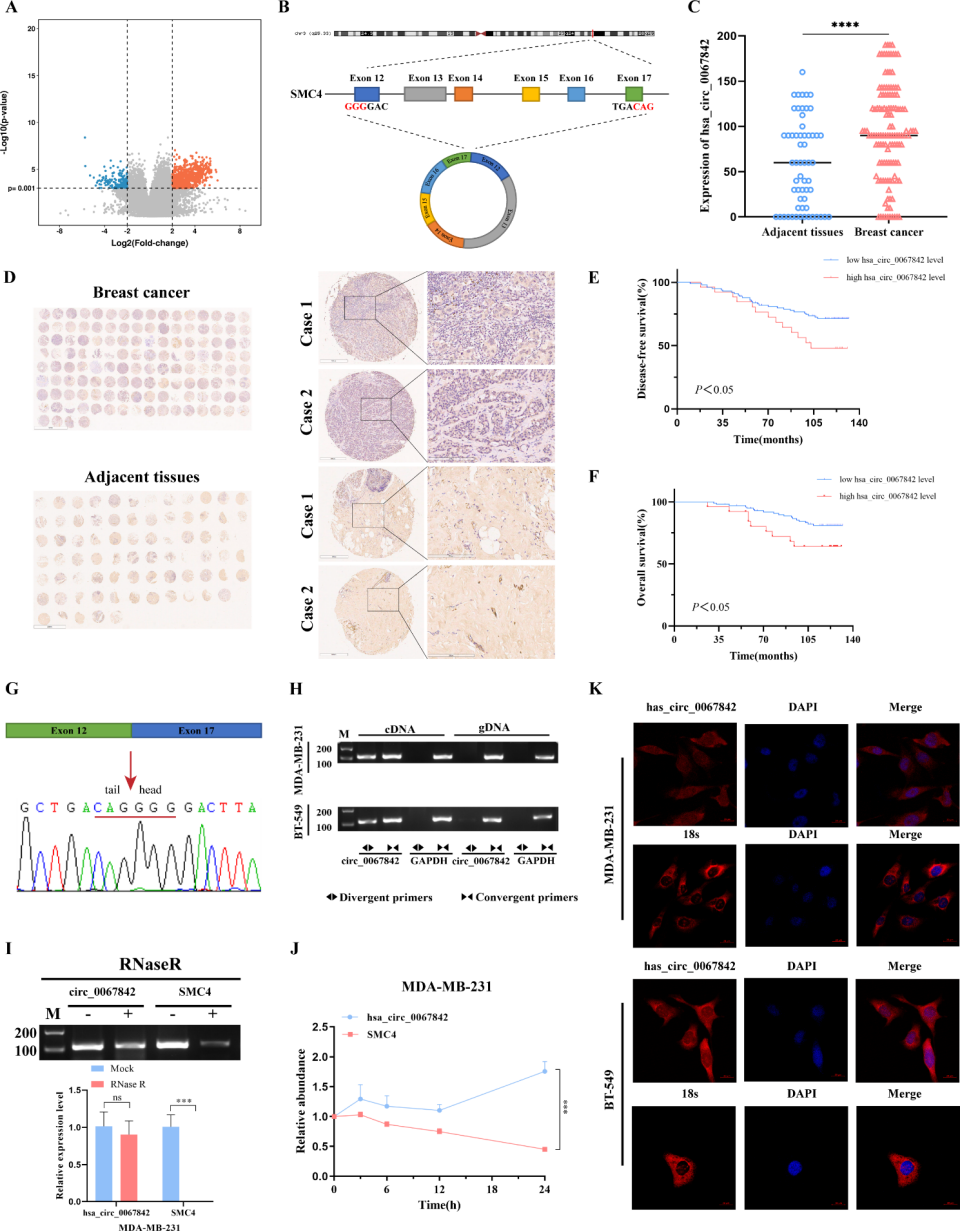
Identification and characterization of hsa_circ_0067842 in BC. **A** Volcano plot showing the expression profiles of circRNAs. **B** Schematic illustrations displaying the genomic loci of hsa_circ_0067842, produced by exons 12 to 17 of the SMC4 gene. **C-D** ISH analysis detecting hsa_circ_0067842 expression (red) in BC tissue (n = 126) and adjacent normal tissues (n = 59). Representative images from C two BC cases and adjacent normal tissues are shown (magnification: ×200). **E-F** Kaplan–Meier analysis of the correlation between hsa_circ_0067842 levels and the disease-free survival or overall survival rate of BC patients. **G** Back-splice junction of hsa_circ_0067842 verified by Sanger sequencing. **H** Convergent or divergent primers were used to validate the existence of hsa_circ_0067842 in BC cells. **I** Total RNAs were treated with RNase R and qRT-PCR was used to detect hsa_circ_0067842 and SMC4. **J** The expression of hsa_circ_0067842 and SMC4 mRNA in BC cells treated with Actinomycin D at the indicated time points was detected by qRT-PCR. **K** FISH assay identifying the subcellular location of hsa_circ_0067842 in BC cell lines. Scale bar, 20 μm. Quantitative data were presented as the mean ± SD, ^***^p < 0.001 and ^****^p < 0.0001

To investigate differentially expressed circRNAs in BC, we performed a circRNA microarray analysis on six pairs of BC tissues and the adjacent normal breast tissues. From this analysis, we identified 129 upregulated and 89 downregulated circRNAs (*P* < 0.001, fold change > 4, length < 1000nt, average signal value > 7, and exon cyclization) (Fig. 1A and additional file 3). Then, we focused on the upregulated circRNAs as they could potentially serve as therapeutic targets or prognostic biomarkers. Among the upregulated circRNAs, we found that hsa_circ_0067842, which derives from the structural maintenance of chromosomes 4 SMC4 (exon 12–17) on chromosome 3 and ultimately forms the mature circRNA with a length of 938 nt (Fig. 1B), was highly expressed in BC as demonstrated by ISH analysis of 126 BC samples compared to 59 adjacent normal tissues (Fig. 1C-D). Although there was no obvious correlation between hsa_circ_0067842 expression and clinicopathological characteristics (Additional file 2: Table S1), its high expression was significantly associated with short disease-free survival (DFS) and overall survival (OS) of BC patients (Fig. 1E-F). In particular, the results of multivariate analysis revealed that hsa_circ_0067842 expression could serve as an independent prognostic factor for BC patients (*P* = 0.011, Table 1). In conclusion, our findings indicated that hsa_circ_0067842 is highly expressed in BC tissues and may serve as a potential prognostic biomarker.

Next, we verified that the characteristic of hsa_circ_0067842, which had relatively high conservative (Additional file 1: Figure S1A), and the putative back-splice junction by Sanger sequencing (Fig. 1G). Since the flanking intronic complementary sequences, particularly ALU repeat elements, have the ability to drive exon cyclization [12], we analyzed the parental SMC4 gene and found complementary ALU sequence, which might serve as a possible mechanism for hsa_circ_0067842 formation (Additional file 1: Figure S1B). Subsequently, we found hsa_circ_0067842 could only be amplified by divergent primers in cDNA, but not in genomic DNA (Fig. 1H). Furthermore, compared with linear SMC4, hsa_circ_0067842 was more resistant to RNase R (Fig. 1I), and its half-life was longer (Fig. 1J). More importantly, we performed RNA-FISH and found that hsa_circ_0067842 was located in both nucleus and cytoplasm (Fig. 1K), suggesting its involvement in BC progression through multiple pathways.

**Table 1:** Univariate and multivariate analyses of the factors correlated with overall survival of 126 BC patients

Variables	Univariate analysis				Multivariate analysis			
	p value	HR	95%CI		p value	HR	95%CI	
			下限	上限			下限	上限
Expression	0.049^*^	2.234	1.003	4.977	0.047^*^	2.277	1.011	5.127
Age	0.078	2.168	0.916	5.129				
Grade stage	0.007^*^	2.855	1.341	6.080	0.011^*^	2.749	1.262	5.990
TNM stage	0.016^*^	2.546	1.191	5.442	0.510	1.531	0.431	5.439
T stage	0.706	1.167	0.524	2.597				
N stage	0.044^*^	2.276	1.022	5.067	0.588	1.438	0.387	5.347

^*^p<0.05

### Hsa_circ_0067842 promotes BC metastasis in vitro

**Fig. 2:**
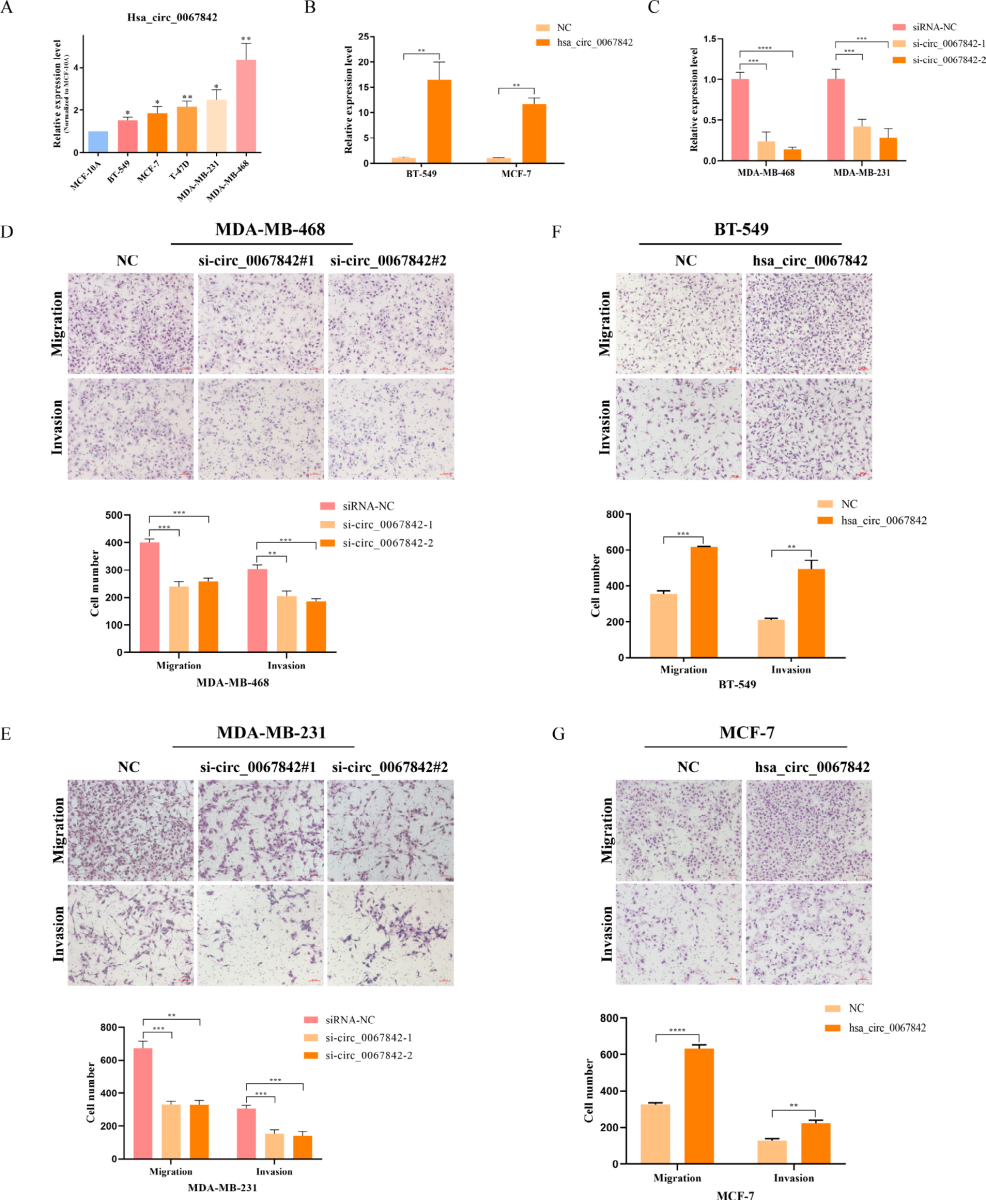
Hsa_circ_0067842 promotes BC metastasis both in vitro. **A** qRT-PCR determining the expression of hsa_circ_0067842 in the normal breast epithelium cell line (MCF-10 A) and BC cell lines (normalized to β-actin). **B-C** qRT-PCR analysis of hsa_circ_0067842 expression in NC plasmid-, hsa_circ_0067842 plasmid-, si-NC-, si-hsa_circ_0067842-1-, and si-hsa_circ_0067842-2-treated BC cells. **D-E** Transwell migration and invasion assays assessing the changes in the migratory and invasive capabilities of MDA-MB-468 and MDA-MB-231 cells after transfection with si-hsa_circ_0067842s. **F-G** Transwell migration and invasion assays assessing the changes in the migratory and invasive capabilities of BT-549 and MCF-7 cells after transfection with NC or hsa_circ_0067842 plasmids. The data are assessed as the mean ± SD, ^*^p < 0.05, ^**^p < 0.01, ^***^p < 0.001 and ^****^p < 0.0001

To determine the role of hsa_circ_0067842 in BC cells, we first examined its expression in six cell lines, including one normal mammary epithelial cell line MCF-10 A and five BC cell lines (MCF-7, BT549, T-47D, MDA-MB-231, and MDA-MB-468). As expected, hsa_circ_0067842 showed a much higher expression in BC cell lines. In an effort to further investigate the oncogenic properties of hsa_circ_0067842, we performed loss-of-function experiments using MDA-MB-468 and MDA-MB-231 cells that had relatively high levels of hsa_circ_0067842, and gain-of-function experiments using MCF-7 and BT-549 cells with relatively low expression. The transfection efficiency was verified after transfecting cells with siRNAs or overexpression plasmids (Fig. 2B-C), and the transfection did not affect the expression of parent gene SMC4 (Additional file 1: Figure S2A). The results of transwell assay showed that knockdown of hsa_circ_0067842 significantly inhibited the migration and invasion capabilities of MDA-MB-468 and MDA-MB-231 cells (Fig. 2D-E). On the other hand, the migration and invasion capabilities of BT-549 and MCF-7 cells were substantially enhanced after hsa_circ_0067842 overexpression. (Figure 2F-G). In addition, the CCK-8 assay and the colony formation assay showed no significant effect of hsa_circ_0067842 on BC cell proliferation (Additional file 1: Figure S2B-S2C). These findings confirmed that has_circ_0067842 could promote BC cells metastasis in vitro.

### Hsa_circ_0067842 regulates the immune escape of BC

**Fig. 3:**
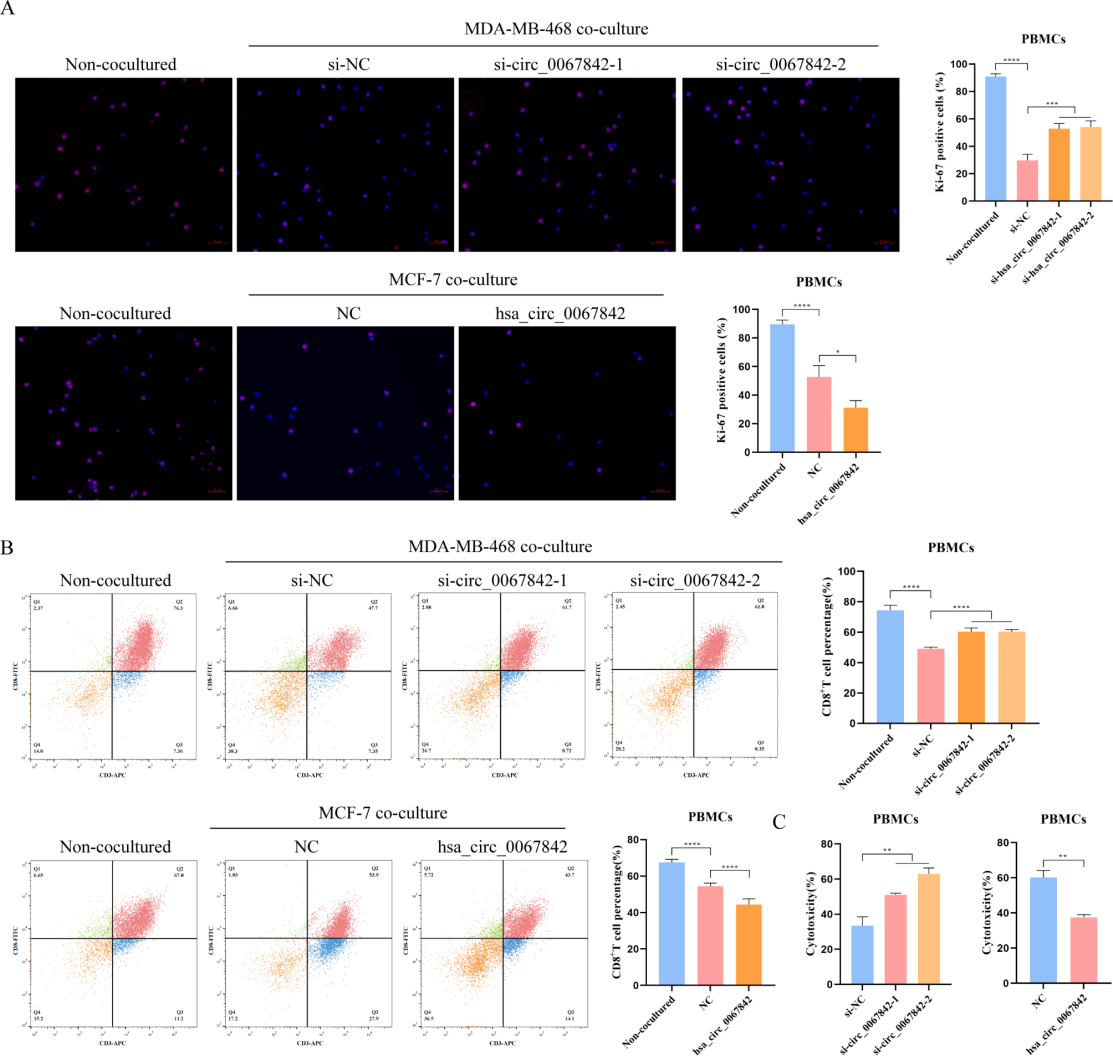
Hsa_circ_0067842 can regulate the immune escape of BC. MDA-MB-468 or MCF-7 cells in different treatment groups were co-cultured with isolated PBMCs. **A** Ki-67 staining was used to observe the PBMCs proliferation. **B** Flow cytometry was used to determine the percentage of CD8^+^ T cells in PBMCs. **C** LDH cytotoxicity assay was used to detect the cytotoxicity of PBMCs. Quantitative data were presented as the mean ± SD, ^*^p < 0.05, ^**^p < 0.01, ^***^p < 0.001 and ^****^p < 0.0001

The immune system has the ability to regulate tumor biology and prevent cancer cells from escaping immune supervision. Numerous studies have demonstrated that immune escape is a contributing factor to the development and progression of cancer, including BC [13]. To verify the role of hsa_circ_0067842 in the immune escape of BC, we co-cultured MDA-MB-468 and MCF-7 cells with PBMCs. As observed, the proliferation of PBMCs was inhibited and the percentage of CD8^+^ T cells was significantly reduced after the co-culture. Interestingly, silencing hsa_circ_0067842 resulted in an increase in PBMC proliferation and a corresponding rise in the percentage of CD8^+^ T cells, while overexpression had the opposite effect (Fig. 3A-B). Furthermore, increased or decreased cytotoxicity of PBMCs was observed in hsa_circ_0067842 knockdown or upregulated cells (Fig. 3C). These results suggested that hsa_circ_0067842 could affect the immune escape of BC.

### Hsa_circ_0067842 promotes the translocation of HuR protein into cytoplasm

**Fig. 4:**
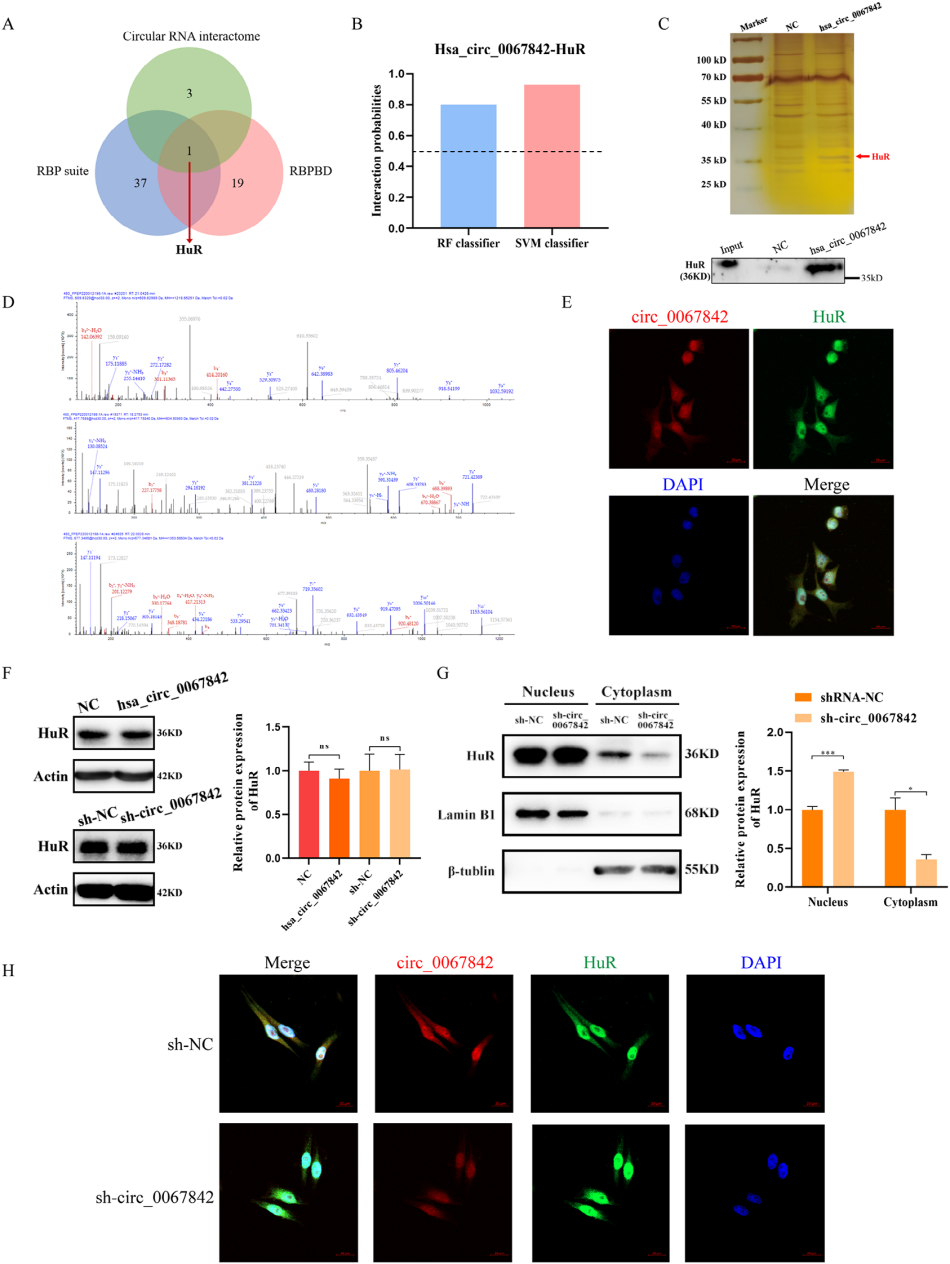
Hsa_circ_0067842 promotes the translocation of HuR protein into the cytoplasm. **A** Venn diagram showing the RBPs interacted with hsa_circ_0067842. **B** Interaction probabilities between HuR and hsa_circ_0067842 predicted by RPISeq (> 0.5 were considered “positive”). **C** Western blot of the proteins from hsa_circ_0067842 pull-down assay, followed by silver staining. **D** Three HuR protein-specific peptides were identified by mass spectrometry. **E** FISH for hsa_circ_0067842 (red) and HuR (green) in BC cells (MDA-MB-468 cell line). DAPI were used to stained for nuclei (blue). Scale bars are 20 μm. **F** The relative expression of HuR was detected by western blot after hsa_circ_0067842 overexpression (MCF-7) or knockdown (MDA-MB-468). **G** After knocking down hsa_circ_0067842, the level of nuclear HuR protein significantly increased, while the cytoplasmic HuR protein obviously decreased. **H** Fluorescence localization analysis of hsa_circ_0067842 and HuR showed that downregulating hsa_circ_0067842 could inhibit the translocation of HuR into the cytoplasm. The data are assessed as the mean ± SD, ^ns^*p* > 0.05, ^*^p < 0.05, and ^***^p < 0.001

Numerous studies have demonstrated that RNA-binding proteins (RBPs) are crucial for cancer progression, and circRNAs may regulate cancer by interacting with RBPs [14, 15]. Based on the above results, we aimed to further investigate whether hsa_circ_0067842 regulates BC progression through interacting with RBP. As indicated by the prediction results from Circular RNA Interactome database, RBPsuite database, and RNA-Binding protein database (RBPDB), RBP HuR was the only protein predicted by all databases (Fig. 4A), and 5 potential binding sites were identified (Additional file 1: Figure S3A). To further examine the interaction between hsa_circ_0067842 and HuR, we used RNA-Protein Interaction Prediction (RPISeq). The scores for RF Classifier and SVM Classifier were 0.8 and 0.93, respectively (Fig. 4B), indicating a high binding probability between hsa_circ_0067842 and HuR. Next, we performed RNA pull down assay to confirm the interaction between hsa_circ_0067842 and HuR, followed by silver staining and mass spectrometry (Fig. 4C). The specifc amino acid sequences of HuR were identified from MS assay (Fig. 4D). Western blot analysis also showed a significant enrichment of HuR in the RNA pull down assay with hsa_circ_0067842 probe (Fig. 4C). We also performed FISH and IF experiments to explore the subcellular localization of hsa_circ_0067842 and HuR, and found that they were co-localized (Fig. 4E). Since overexpression or deletion of hsa_circ_0067842 did not obviously change the expression of HuR (Fig. 4F), and HuR is usually considered as a nuclear shuttle protein [16], we then wondered whether hsa_circ_0067842 played a role in regulating the translocation of HuR protein. Subcellular fraction assay showed that knocking down hsa_circ_0067842 decreased the expression of cytoplasmic HuR and increased its expression in the nucleus (Fig. 4G). Further FISH and IF experiments confirmed that silencing of hsa_circ_0067842 could inhibit HuR shuttling from nucleus to cytoplasm (Fig. 4H). These data suggest that hsa_circ_0067842 can interact with HuR to influence its distribution in cytoplasm.

### Hsa_circ_0067842 increases mRNA stability of CMTM6 which then promotes the migration and invasion of BC cells

**Fig. 5:**
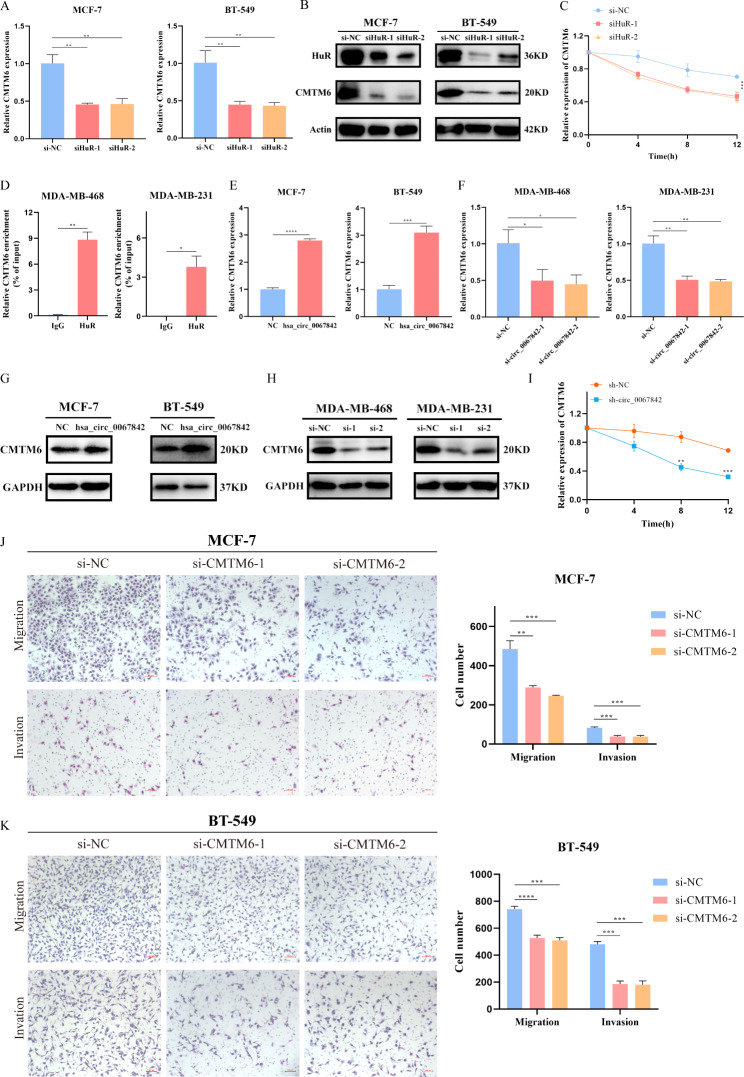
Hsa_circ_0067842 increases mRNA stability of CMTM6 by regulating HuR and CMTM6 promotes migration and invasion of BC cells. **A-B** The expression of CMTM6 after knocking down HuR was verified by qRT-PCR and western blot. **C** RNA stability was evaluated using Actinomycin D in MCF-7 cell, and the degradation rates of the CMTM6 mRNA were measured at appointed time. **D** RNA immunoprecipitation experiments were performed in MDA-MB-468 and MDA-MB-231 cells and the coprecipitated RNA was subjected to qRT-PCR for CMTM6. **E-H** The expression of CMTM6 after altering hsa_circ_0067842 was verified by qRT-PCR and western blot. **I** RNA stability was evaluated using Actinomycin D in MDA-MB-468, and the degradation rates of the CMTM6 mRNA were measured at appointed time. **J-K** Transwell migration and invasion assays assessing the changes in the migratory and invasive capabilities of MCF-7 and BT-549 cells after transfection with si-CMTM6s. Values are shown as the mean ± SD of the mean from three independent experiments. ^*^P < 0.05, ^**^P < 0.01, ^***^P < 0.001, ^****^P < 0.0001

Our results highlighted the importance of hsa_circ_0067842 in BC metastasis and immune escape. Thus, we next wanted to explore the downstream target genes and possible mechanisms involved, with a focus on the interaction between hsa_circ_0067842 and HuR. HuR has been reported as a key regulator in disease pathology via promoting mRNA stability [17]. Recent research has shown that HuR can up-regulate CMTM6 expression in a variety of cancers, and this positive correlation was of interest to us [18]. To explore the relationship between HuR and CMTM6, we silenced HuR in MCF-7 and BT-549 cells. The results of qRT-PCR and western blot showed that the mRNA and protein expression of CMTM6 significantly decreased with the reduction of HuR (Fig. 5A and B). Moreover, actinomycin D treatment showed that HuR could affect the mRNA stability of CMTM6 (Fig. 5C). Additionally, RIP assay was performed to confirm the interaction between HuR and CMTM6, which showed that HuR indeed bound to CMTM6 (Fig. 5D). Based on the results that CMTM6 transcript stability increased by HuR binding, we wanted to further investigate the potential impact of hsa_circ_0067842 on CMTM6 abundance. Upon hsa_circ_0067842 overexpression in MCF-7 and BT-549 cells, the CMTM6 mRNA level increased (Fig. 5E). Consistently, knockdown of hsa_circ_0067842 led to a significant decrease in CMTM6 mRNA expression (Fig. 5F). The upregulation of CMTM6 by hsa_circ_0067842 overexpression was also confirmed by immunoblotting (Fig. 5G-H). In addition, the stability of CMTM6 mRNA was examined in sh-NC or sh-hsa_circ_0067842 stable cell lines after actinomycin D treatment. The results showed that the half-life of CMTM6 mRNA was significantly reduced in sh-hsa_circ_0067842 cells compared to control (Fig. 5I). Taken together, our results showed that hsa_circ_0067842 regulated BC progression through interacting with HuR, which mediated the stabilization of CMTM6 mRNA. We also explored the biological functions of CMTM6 in BC. Knockdown of CMTM6 (Additional file 1: Figure S3C) resulted in suppressed migration and invasion of BC cells (Fig. 5J K), suggesting that hsa_circ_0067842 may act as an oncogenic factor in BC cells by targeting CMTM6.

### Hsa_circ_0067842 inhibits the degradation of PD-L1 via HuR/CMTM6 axis

**Fig. 6:**
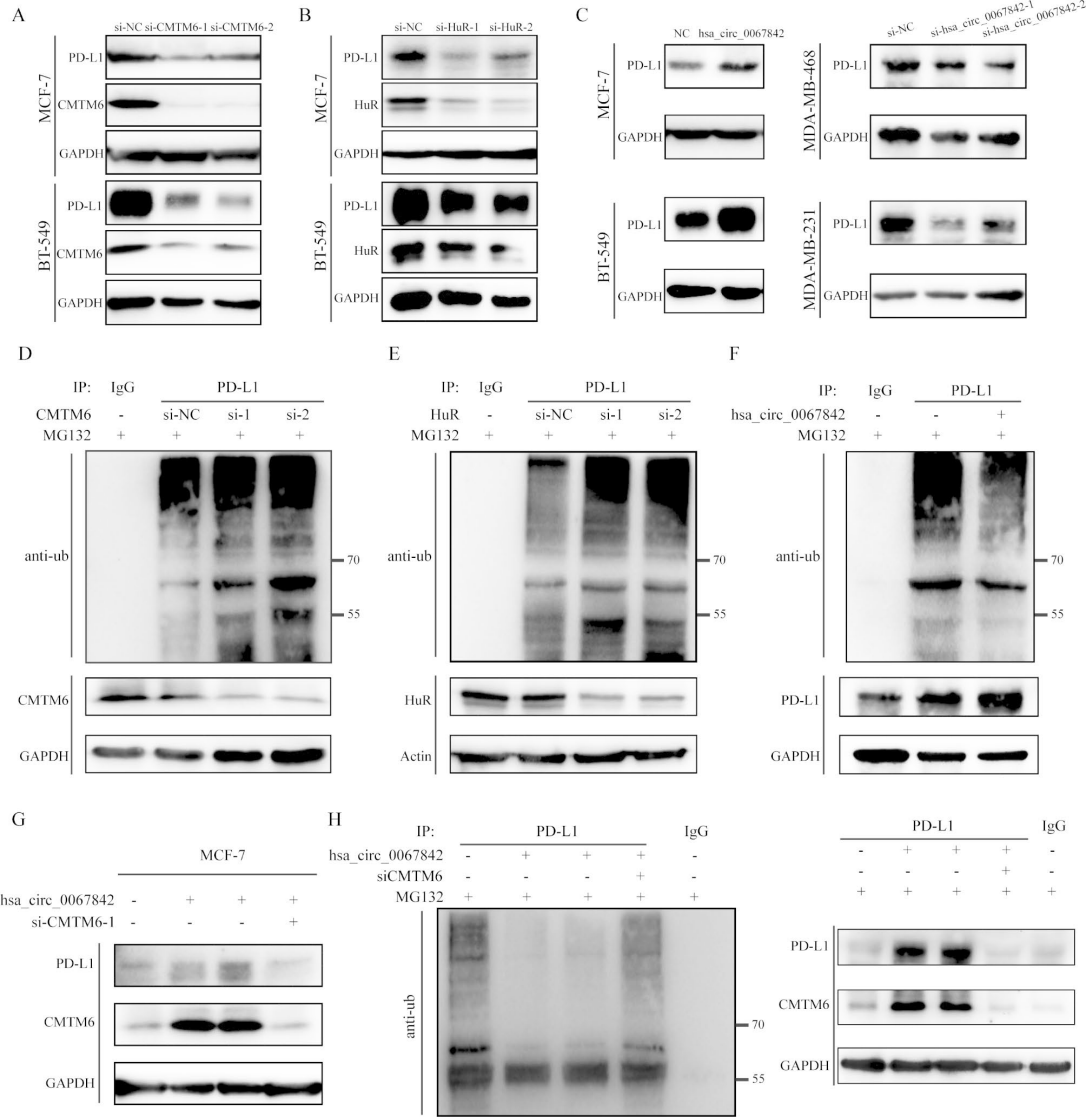
Hsa_circ_0067842 inhibits the degradation of PD-L1 via HuR/CMTM6 axis. **A** Western blot assays of CMTM6 expression in si-NC- or si-CMTM6-expressing MCF-7 or BT-549 cells. **B** Western blot assays of CMTM6 expression in si-NC- or si-HuR-expressing MCF-7 or BT-549 cells. **C** Western blot assays of CMTM6 expression in hsa_circ_0067842- or si-hsa_circ_0067842-expressing BC cells. **D** Ubiquitination level of PD-L1 in MCF-7 cell was detected via immunoprecipitation and western blot after knocking down CMTM6. **E** Ubiquitination level of PD-L1 in MCF-7 cell was detected via immunoprecipitation and western blot after knocking down HuR. **F** Ubiquitination level of PD-L1 in MCF-7 cell was detected via immunoprecipitation and western blot after overexpressing hsa_circ_0067842. **G** Protein expression of PD-L1 and CMTM6 was detected via western blot. **H** Ubiquitination level of PD-L1 in MCF-7 cell was detected via rescue experiments

CMTM6, as a master regulator of PD‑L1, has been reported to inhibit the degradation of PD-L1 through deubiquitination [19–21]. Therefore, we hypothesized that hsa_circ_0067842 could play a role in PD-L1 degradation via HuR/CMTM6 axis. We first analyzed the protein expression and ubiquitination level of PD-L1 in response to CMTM6 knockdown. Consistent with previous studies, the degradation of PD-L1 was significantly decreased in CMTM6-depleted cell (Fig. 6A-D). Since HuR could stabilize CMTM6, we then examined the effects of HuR knockdown on PD-L1 protein stability, and PD-L1 degradation was also suppressed (Fig. 6B-E). Moreover, the PD-L1 protein level was positively correlated with hsa_circ_0067842 expression (Fig. 6C). The ubiquitination level of PD-L1 decreased after hsa_circ_0067842 upregulation (Fig. 6F), and this effect was largely rescued by CMTM6 knockdown (Fig. 6G-H). In conclusion, our results demonstrated that hsa_circ_0067842 could regulate PD-L1 via the HuR/CMTM6 axis.

### CMTM6 knockdown attenuated the effects of hsa_circ_0067842 overexpression on BC metastasis and immune escape

**Fig. 7:**
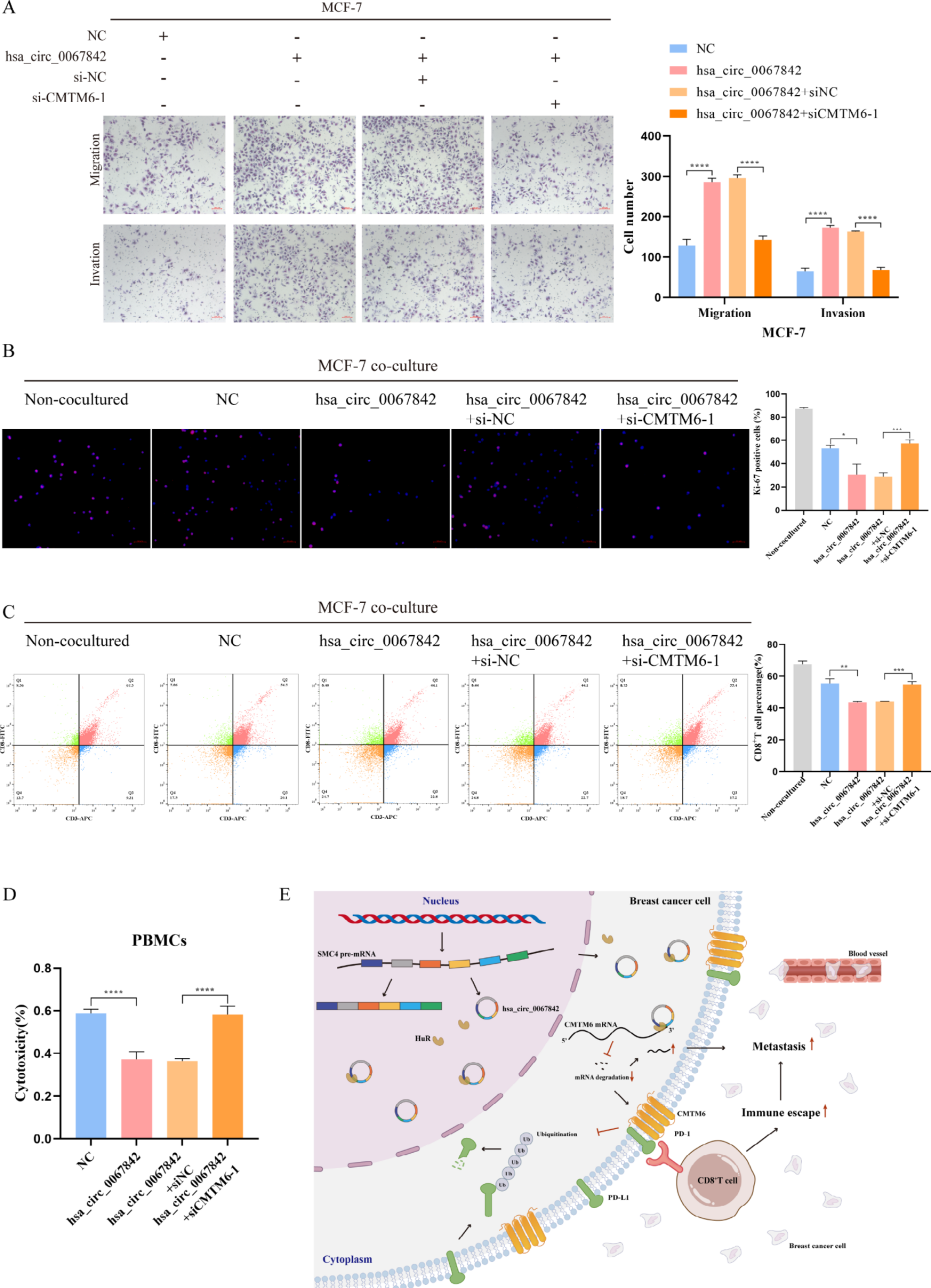
CMTM6 knockdown attenuated the promotion of overexpressing hsa_circ_0067842 on the metastasis and immune escape of BC. **A** Transwell migration and invasion assays assessing the changes in the migratory and invasive capabilities of MCF-7 in rescue experiments. **B** Ki-67 staining was used to observe the PBMCs proliferation in rescue experiments. **C** Flow cytometry was used to determine the percentage of CD8^+^ T cells in rescue experiments. **D** LDH cytotoxicity assay was used to detect the cytotoxicity of PBMCs in rescue experiments. **E** Mechanism of hsa_circ_0067842 in metastasis and immune escape of BC. The experiments were performed three times, and data are represented as mean ± SD, ^ns^*P* > 0.05, ^**^P < 0.01, ^***^P < 0.001, ^****^P < 0.0001

Rescue experiments were further conducted to confirm the involvement of CMTM6 in the promotion of BC metastasis by hsa_circ_0067842. The amount of CMTM6 was first measured (Additional file 1: Figure S3D). As expected, CMTM6 knockdown partially reversed the effect of hsa_circ_0067842 overexpression on BC migration and invasion (Fig. 7A). Moreover, given the interplay between hsa_circ_0067842 and CMTM6, as well as the regulation of CMTM6 on PD-L1, we then investigated the role of CMTM6/PD-L1 axis in hsa_circ_0067842-regulated immune escape. We transfected NC and hsa_circ_0067842 plasmids into MCF-7 cells to upregulate hsa_circ_0067842 expression, followed by simultaneous treatment with si-CMTM6. The transfected MCF-7 cells were then co-cultured with PBMCs. We found that CMTM6 knockdown partially reversed the inhibitory effect of hsa_circ_0067842 on PBMCs proliferation (Fig. 7B), CD8^+^ T cells percentage (Fig. 7C), and PBMCs cytotoxicity (Fig. 7D). These results indicated that hsa_circ_0067842 promoted the metastasis and immune escape of BC by regulating CMTM6.

## Discussion

The mortality rate of BC is slowly decreasing, suggesting some progress in the diagnosis and treatment of BC [1]. However, the prognosis of BC patients is still poor, especially for those with metastasis, which is still incurable [22]. Current research has found that the immunosuppressive factors play a role in promoting recurrence and metastasis of post-surgery BC patients, and immunotherapy is a promising treatment for advanced or metastatic BC [23–25]. Therefore, to fully understand the underlying molecular mechanism of BC metastasis and to identify new therapeutic targets is still the top priority of current research.

CircRNAs, a unique kind of non-coding RNAs, play a crucial role in disease development, especially in various cancers including BC metastasis, thus gaining increasing attention from researchers. For example, Wang et al. [26] showed that circPRKCI could promote TNBC proliferation and metastasis by upregulating WBP2 expression and activating PI3K/AKT signaling pathway. Similarly, another study demonstrated that circACTN4 regulates the expression of oncogene MYC, promoting BC progression through interacting with FUBP1 [27]. In this study, we identified a novel, highly expressed circRNA hsa_circ_0067842 in BC tissues by microarray analysis. The circRNA originated from the parental gene SMC4. Our analysis of GTEx data showed a lower SMC4 expression in mammary tissues, suggesting that hsa_circ_0067842 may play a crucial role in BC progression (Additional file 1: Figure S1C). Through analyzing the relationship between hsa_circ_0067842 and prognosis of BC patients, we found that higher hsa_circ_0067842 expression was associated with shorter DFS and OS, indicating a worse prognosis. Moreover, hsa_circ_0067842 was identified as an independent prognostic factor for BC. Our hypothesis was further supported by loss- and gain-of-function experiments, which showed that hsa_circ_0067842 promoted the migration and invasion capabilities of BC cell. Subsequently, we investigated the role of hsa_circ_0067842 in BC immune escape. PBMCs were co-cultured with hsa_circ_0067842 knockdown MDA-MB-468 cells and hsa_circ_0067842 overexpressed MCF-7 cells. The results showed that silencing or overexpressing hsa_circ_0067842 promoted or inhibited PBMC proliferation, respectively, along with a corresponding increase or decrease in the percentage of CD8^+^ T cells and PBMC cytotoxicity. In summary, our study revealed the importance of hsa_circ_0067842 in BC metastasis and immune escape, establishing it as a potential prognostic and therapeutic target for metastatic BC.

Since the function of circRNA is often related to its localization [28], we first assessed the subcellular localization of hsa_circ_0067842 and found that it was present in both nucleus and cytoplasm, suggesting that it may have multiple biological functions. Recent studies have revealed that circRNAs can interact with RBPs, providing a new avenue of exploration [29, 30]. For example, circNSUN2, an abnormally expressed circRNA in colorectal carcinoma (CRC) tissues, could regulate the stability of HMGA2 mRNA by binding to IGF2BP2, resulting in cancer progression [31]. Hence, we investigated whether hsa_circ_0067842 could bind to any specific protein and modulate its function. Interestingly, HuR was the only protein predicted to bind to hsa_circ_0067842, which was confirmed by RNA pull-down and FISH experiments. We also identified potential binding regions between hsa_circ_0067842 and HuR by predicting the secondary structure of hsa_circ_0067842 (Additional file 1: Figure S3B), but these predictions need further verification.

HuR, a nuclear shuttle protein, is known to bind to a subset of mRNAs and regulate their stability and localization [17, 32–34]. In this study, we found that hsa_circ_0067842 did not affect the HuR expression, so we hypothesized that hsa_circ_0067842 could regulate the translocation of HuR, which was verified by subcellular component isolation assay. Further colocalization assays confirmed the effect of hsa_circ_0067842 on the translocation of HuR from nucleus to cytoplasm. Next, we aimed to investigate the potential target mRNA of HuR. Previous studies have shown that HuR regulates the stability of CMTM6, which can promote tumor metastasis through various mechanisms such as affecting epithelial–mesenchymal transition (EMT), activating immune cells, and regulating signaling pathways [35–37]. Therefore, we explored whether hsa_circ_0067842 could regulate tumor metastasis by affecting the stability of CMTM6 through binding to HuR. qRT-PCR and western blot results showed that the mRNA and protein levels of CMTM6 changed with the alteration of hsa_circ_0067842. The stability of CMTM6 decreased in sh-hsa_circ_0067842 stable cell line treated with actinomycin D. Moreover, silencing CMTM6 inhibited BC cell migration and invasion, which partially blocked hsa_circ_0067842-induced BC metastasis. These results indicated that hsa_circ_0067842 affected BC metastasis by regulating CMTM6 through HuR. Further studies are needed to fully understand the specific mechanism of CMTM6’s role in BC metastasis.

It is well known that PD-L1 can promote immune escape in multiple human cancers by interacting with programmed death-1 (PD-1) [38, 39]. In this study, we found that hsa_circ_0067842 can affect the expression of PD-L1. By silencing or overexpressing hsa_circ_0067842, the protein level of PD-L1 changed accordingly. Thus, we then investigated the mechanism of hsa_circ_0067842 regulation of PD-L1. Previous studies have reported that CMTM6 is a key regulator of PD-L1 expression by preventing the ubiquitination of PD-L1 and inhibiting its degradation [19–21]. Given the regulatory relationships betweem hsa_circ_0067842, HuR and CMTM6, we sought to determine whether hsa_circ_0067842 affects the ubiquitination of PD-L1 through HuR/CMTM6 axis. As expected, we found that the ubiquitination level of PD-L1 decreased after overexpression of hsa_circ_0067842 compared to the control. Furthermore, rescue assays confirmed that hsa_circ_0067842 could regulate the expression of PD-L1 by affecting the ubiquitination of PD-L1, which is mediated by CMTM6. Considering the role of PD-L1 in immune response, we further investigated whether the involvement of hsa_circ_0067842 in immune escape was through HuR/CMTM6/PD-L1 pathway. The results showed that silencing CMTM6 enhanced the degradation of PD-L1, thus partially reversing the inhibitory effect of hsa_circ_0067 842 overexpression on immune function. Overall, our findings revealed a novel mechanism in BC progression where hsa_circ_0067842 promotes immune escape by interacting with HuR, leading to increased stability of CMTM6 and decreased ubiquitination of PD-L1. In recent years, anti-PD1/PD-L1 agents have been used in the treatment of BC, especially in metastatic BC or TNBC, often in combination with conventional treatments [40, 41]. Hence, hsa_circ_0067842 may serve as a potential therapeutic target for metastatic BC, though further studies are needed to confirm this.

In summary, we identified and a new circRNA, hsa_circ_0067842, which was highly expressed in BC tissues. The high expression of hsa_circ_0067842 was associated with poor prognosis of BC patients, making it a potential prognostic factor. Our functional assays showed hsa_circ_0067842 played an oncogenic role in BC by regulating BC metastasis and immune escape. The mechanism behind hsa_circ_0067842’s role in BC was identified to be through transporting nuclear HuR protein into the cytoplasm, leading to the stabilization of CMTM6 mRNA and the upregulation of PD-L1 in BC cells (Fig. 7E). Overall, the hsa_circ_0067842/HuR/CMTM6/PD-L1 axis is a promising target for further exploration regarding its potential applications in BC prognosis and treatment.

## Conclusion

Our study identified a novel circRNA hsa_circ_0067842 highly expressed in BC tissues, serving as an independent prognostic factor for BC. To our knowledge, this is the first study to demonstrate that hsa_circ_0067842 affects metastasis and immune escape of BC through the HuR/CMTM6/PD-L1 axis, providing new ideas for BC research and potential molecular targets for the prognosis and treatment of BC.

## Materials and methods

### BC cell lines and cell culture

The HEK-293T cell line, human mammary epithelial cell line MCF-10 A, and human breast cancer (BC) cell lines MDA-MB-231, MDA-MB-468, MCF-7, and BT549 were purchased from the Cell Bank, Type Culture Collection Committee, Chinese Academy of Sciences (Shanghai, China). Cell lines were maintained under standard media and conditions. MDA-MB-231, MDA-MB-468, MCF-7, and HEK293T were cultured in Dulbecco’s modified Eagle’s medium (DMEM, Gibco, Rockville, MD, USA) supplemented with 10% fetal bovine serum (FBS, Gibco, Rockville, MD, USA) and 1% antibiotic solution. The culture medium for BT549 was Roswell Park Memorial Institute (RPMI, Gibco)1640 medium (Gibco, Rockville, MD, USA) supplemented with 10% FBS and 1% antibiotic solution. MCF-10 A was cultured in DMEM/F12 (Macgene, Beijing, China) medium supplemented with 5% horse serum, 10 μg/ml insulin (Macgene, Beijing, China), 20 ng/ml epidermal growth factor, 100 ng/ml cholera toxin (Macgene, Beijing, China), and 0.5 μg/ml hydrocortisone (Macgene, Beijing, China). All cell lines were cultured at 37° C, 5% CO_2_ in a humidified cell culture incubator.

### CircRNA microarray analysis

Total RNA from six sample pairs (BC tissue and adjacent normal tissue) was extracted using TRIzol reagent (Invitrogen Life Technology, Waltham, MA, USA) and subjected to quality inspection by NanoDrop one spectrophotometer (ThermoFisher, MA, USA). The microarray analysis was conducted by Sinotech Genomics Corporation. Firstly, RNA samples were synthesized to biotinylated cRNAs for the Sino Human ceRNA array V3.0. The Agilent Bioanalyzer 2100 (Agilent technologies, USA) was used to examine the RNA integration of total RNA. Next, the biotinylated cRNAs hybridized on slides were scanned by an Agilent Microarray Scanner (Agilent technologies, USA) and then analyzed by the Feature Extraction software 10.7 (Agilent technologies, USA). Finally, the differentially expressed genes were obtained and heatmaps were plotted using the R software package.

### Tissue microarray (TMA) and in situ hybridization (ISH)

A specific hsa_circ_0067842 probe was designed to detect the expression of hsa_circ_0067842 by ISH in tissues, which contained 126 paraffin-embedded BC samples and 59 paired adjacent normal samples. The tissues of BC patients were obtained from OUTDO BIOTECH (Shanghai, China). The thickness of all tissue slides was 4 μm and the diameter of each tissue core was 1.5 mm. Next, the staining and expression of hsa_circ_0067842 were analyzed using Nikon microscope by quantitative scanning. The relative expression of hsa_circ_0067842 was expressed by the ISH score which was calculated using the positive staining intensity score multiplied by the proportion of positive staining cells.

### Quantitative reverse transcription-polymerase chain reaction (qRT-PCR)

The total RNA was isolated from cultured cells using TRIzol reagent (Invitrogen Life Technology, Waltham, MA, USA). First-strand cDNA was synthesized using the Evo M-MLV RT Reagent Kit (AG, Hunan, China). SYBR Green Pro Taq HS Premix Reagent Kit (AG, Hunan, China) was used to prepare the 10 μL qPCR reaction. β-Actin was used as the reference gene for normalization. The qPCR primers are listed in Additional file 2: Table S2.

### RNase R treatment, actinomycin D assay, and RNA stability measurement

Total RNA was extracted using TRIzol reagent (Invitrogen Life Technology, Waltham, MA, USA). 5ug of RNA was incubated with RNase R at 37 °C for 1 h, and an equal amount of RNA was incubated without RNase R as a control, which were then used for qRT-PCR. Actinomycin D assay was performed by adding 2 μg/mL actinomycin D (MCE, USA) to MDA-MB-231 cell, followed by analyzing the stabilities of hsa_circ_0067842 and linear SMC4 mRNA using qRT-PCR. The stability analysis of CMTM6 was same as that of hsa_circ_0067842, but the actinomycin concentration was 10 μg/mL.

### RNA fluorescence in situ hybridization (FISH)

Cy3-labeled hsa_circ_0067842, 18 S rRNA probes, and the Fluorescent In situ Hybridization Kit were purchased from GenePharma (Shanghai, China). According to the manufacturer’s instructions, cells on coverslips were fixed with 4% paraformaldehyde, treated with 0.5% Triton, incubated with the specific probe overnight, and then stained with DAPI (4′,6-diamidino-2-phenylindole). The cells were imaged using a confocal microscope (Zeiss, Germany). The sequences of the hsa_circ_0067842 probe and 18 S rRNA probe for FISH are listed in Additional file 2: Table S3.

### Cell transfection

The short interfering RNAs against Hsa_circ_0067842 (siRNAs) (si-circ_0067842-1, si-circ_0067842-2), HuR siRNA (siHuR-1, siHuR-2), CMTM6 siRNA (siCMTM6-1, siCMTM6-2), and corresponding negative controls (si-NC) were purchased from Shanghai GenePharma (Shanghai, China). The Hsa_circ_0067842 pLCDH-ciR (pLCDH -hsa_circ_0067842) and corresponding negative controls (pLCDH-NC) were obtained from ViGene bioscience (Jinan, Shandong, China). These siRNAs and plasmids were transfected into cells using Lipofectamine 2000 (Invitrogen, CA, USA). The sequences of the siRNAs are listed in Additional file 2: Table S5.

### Lentivirus packaging and infection

The si-hsa_circ_0067842-2 sequence was cloned into lentiviral vector pLent-U6-shRNA-GFP-Puro by ViGene bioscience (Jinan, Shandong, China), and the empty vector was used as control. HEK-293T cells were transfected with the viral plasmids and packaging plasmids to make virus. The supernatant was collected at 72 h after transfection and incubated with BC cells for transduction. After 2 weeks of puromycin selection at 1 μg/ml, the BC cells were cultured in batch for follow-up test.

### Colony formation assay

MDA-MB-231 (3 × 10^3^) cells and MCF-7 (2 × 10^3^) cells were seeded in 6-well plates. After 7 days, the cell colonies were fixed with 4% paraformaldehyde (servicebio, Wuhan, China) for 30 min, stained with 0.1% crystal violet (Solarbio, Beijing, China) for 30 min and counted.

### Cell viability assay

The transfected MDA-MB-231 (4 × 10^3^) and MCF-7 (3 × 10^3^) cells were harvested and seeded into 96-well plates. 10ul of Cell Counting Kit 8 (CCK-8, Bestbio, Shanghai, China) solution was added into each well, followed by incubation at 37 °C for 1 h. Cell viability was measured every 24 h by SpectraMax i3X (Molecular Devices, USA) at 450 nm wavelength.

### Cell migration and invasion assays

Cell migration and invasion assays were performed using the chamber inserts with 8-μm pore size (Corning, NY, USA), precoated with or without 60 μl Matrigel (BD Biosciences, USA). MDA-MB-231 cells (5 × 10^4^ for migration / 1 × 10^5^ for invasion) and MCF-7 cells (4 × 10^4^ for migration / 8 × 10^4^ for invasion) were resuspended in 200 μl serum-free medium and seeded into upper chamber, and 600ul of medium supplemented with 20% FBS was added to the lower chamber. After 30 h of incubation, the cells migrated to lower filters were fixed with 4% paraformaldehyde, stained with Giemsa’s stain, imaged under an inverted microscope (Zeiss, Germany), and analyzed with ImageJ software.

### Isolation of peripheral blood mononuclear cells (PBMCs)

Peripheral venous blood (2–4 mL) was collected from healthy subjects, and the PBMCs were isolated from blood using the Ficoll density separation method. PBMCs were cultured in RPIM-1640 medium and activated by Human CD3/CD28 T-Activator (Stemcell, Canada) for 1 week.

#### Co‑culture and lactate dehydrogenase (LDH) cytotoxicity assays

MDA-MB-468 or MCF-7 cells were seeded into 12-well plates at 5 × 10^4^cells/well. After 24 h, the activated PBMCs were added to co-culture with BC cells at a ratio of 5:1. After 48 h of co-culture, PBMC cytotoxicity was measured using the LDH cytotoxicity detection kit (Abbkine, Wuhan, China).

### Flow cytometry

The co-cultured PBMCs were stained with anti-CD3 and anti-CD8 antibodies, and then analyzed with flow cytometry on Cytoflex platform (Beckman, USA). FlowJo software was used for gating CD8^+^ T cells, which were positive for CD3 and CD8.

#### Western blot

Cells were harvested and lysed with Western/IP lysis buffer (Beyotime, Shanghai, China) supplemented with protease inhibitor Phenylmethanesulfonyl fluoride (PMSF, Beyotime, Shanghai, China). Protein samples were denatured, loaded onto SDS-PAGE, transferred to 0.45 mm polyvinylidene fluoride (PVDF) membranes (Merck-Millipore, Darmstadt, Germany), and blocked with 5% BSA (Bovine Serum Albumin) blocking solution. After incubated with primary antibodies at 4 °C overnight and corresponding secondary antibodies at room temperature for 1 h, the membranes were analyzed with HRPO-induced chemiluminescence. The antibodies used in western blot are listed in Additional file 2: Table S4.

#### RNA pull-down assay and mass spectrometry (MS)

A biotin-labeled probe targeting the junction site of hsa_circ_0067842 was synthesized by General Biol (Anhui, China), and oligo probe was used as control. According to the manufacturer’s protocol, the interaction between hsa_circ_0067842 and RNA binding protein was measured using Pierce Magnetic RNA-Protein Pull-Down Kit (Thermo Fisher Scientific, USA). Briefly, protein extracted from MDA-MB-231 cells was incubated with biotin-labeled probes and streptavidin magnetic beads at 4 °C. Then, the circRNA-associated proteins were eluted, separated by SDS-PAGE, and visualized with Silver Staining Kit (Thermo Fisher Scientific, USA). The retrieved proteins were then subjected to mass spectrometry (MS) analysis (Novogene, Tianjin, China).

#### Immunofluorescence (IF)

The BC cells co-cultured with PBMCs were seeded on poly-lysine-coated glass coverslips and fixed for IF staining. The coverslips were blocked with 10% BSA and incubated with anti-Ki-67 antibody at 4 °C overnight. Subsequently, cells were incubated with Alexa Fluor 555-labeled anti-rabbit secondary antibody for 1 h at room temperature. Finally, cells were stained with DAPI for 20 min, followed by image acquisition.

### PD‑L1 ubiquitination detection

BC cells in different treatment groups were lysed with 0.3% NP-40 lysis buffer. The lysates were incubated with IgG or PD-L1 at 4 °C overnight. Next, protein A/G agarose beads (Novex, Oslo, Norway) were added and incubated at 4 °C for 4 h. After centrifugation, the supernatant was collected for ubiquitination level analysis via western blot.

### Statistical analysis

All data analyses were performed using GraphPad Prism V9.0 (GraphPad prism, Inc., La Jolla, CA, United States). Student’s t test, χ^2^, or one-way ANOVA test were used to test the differences between groups. The results were expressed as mean ± standard (SD). P value < 0.05 was considered statistically significant.

### Electronic supplementary material

Below is the link to the electronic supplementary material.

**Additional file 1: Figure S1**. The genomic locus and characteristics of hsa_circ_0067842. **A** Schematic diagram showing the conservation of hsa_circ_0067842. **B** Schematic diagram showing the genomic locus and the flanking introns with ALU sequence of hsa_circ_0067842 (upper). Complementary ALU sequence (FLAM_A-AluJb and FLAM_A-AluSg) in the flanking introns of SMC4 exon 12 to exon 17 (lower). **C** Expression of SMC4 across diverse normal human tissues from GTEx (https://www.gtexportal.org/home/index.html); **Figure S2**. The effects of hsa_circ_0067842 on proliferation of BC cells in vitro. **A** qRT-PCR detecting the expression of SMC4 in BC cells after transfection of the NC or hsa_circ_0067842 plasmid and siRNAs si-NC, si-hsa_circ_0067842-1, si-hsa_circ_0067842-2. **B** CCK-8 assays in hsa_circ_0067842-overexpressing MCF-7 or hsa_circ_0067842-depleted MDA-MB-231 cells. **C** Colony formation assays in hsa_circ_0067842-overexpressing MCF-7 or hsa_circ_0067842-depleted MDA-MB-231 cells; **Figure S3**. The relationship among hsa_circ_0067842, HuR, and CMTM6. **A** The binding sites of hsa_circ_0067842 and HuR predicted by RBP suite. **B** Secondary structure of hsa_circ_0067842 and high probable binding regions. **C** The transfection efficiency of si-CMTM6s in BT-549 and MCF-7 cells was verified by qRT-PCR and western blot. **D** The expression of CMTM6 in rescue experiments was assessed by qRT-PCR and western blot

**Additional file 2: Table S1**. Correlation between hsa_circ_0067842 expression and clinicopathological characteristics; **Table S2**. Sequences of primers used for qRT-PCR in this study; **Table S3**. Sequences of probes used in this study; **Table S4**. Antibodies for Immunoblotting in this study; **Table S5**. Oligonucleotides used in the study

**Additional file 3**

## Data Availability

The datasets used and/or analyzed during the current study are available from the corresponding authors on reasonable request.

## List of abbreviations

BC: Breast cancer
CCK-8: Cell Counting Kit-8
cDNA: Complementary DNA
circRNAs: Circular RNAs
DAPI: 4′,6-diamidino-2-phenylindole
DFS: Disease-free survival
EMT: Epithelial–mesenchymal transition
FISH: Fuorescence in situ hybridization
gDNA: Genomic DNA
IF: Immunofluorescence
MS: Mass spectrometry
OS: Overall survival
PBMCs: Peripheral blood mononuclear cells
PD-1: Programmed death-1
PD-L1: Programmed death ligand-1
RBPs: RNA-binding proteins
RIP: RNA immunoprecipitation
siRNA: Small interfering RNA
TNBC: Triple-negative breast cancer
