# Supplementary material for: Circular RNA hsa_circ_0067842 facilitates tumor metastasis and immune escape in breast cancer through HuR/CMTM6/PD-L1 axis
Source: Biol Direct. 2023 Aug 18;18:48. doi: 10.1186/s13062-023-00397-3 (PMC10436663; doi:10.1186/s13062-023-00397-3)
Supplement: Supplementary file 1 — Additional file 1: Figure S1. The genomic locus and characteristics of hsa_circ_0067842. A Schematic diagram showing the conservation of hsa_circ_0067842. B Schematic diagram showing the genomic locus and the flanking introns with ALU sequence of hsa_circ_0067842 (upper). Complementary ALU sequence (FLAM_A-AluJb and FLAM_A-AluSg) in the flanking introns of SMC4 exon 12 to exon 17 (lower). C Expression of SMC4 across diverse normal human tissues from GTEx (https://www.gtexportal.org/home/index.html); Figure S2. The effects of hsa_circ_0067842 on proliferation of BC cells in vitro. A qRT-PCR detecting the expression of SMC4 in BC cells after transfection of the NC or hsa_circ_0067842 plasmid and siRNAs si-NC, si-hsa_circ_0067842-1, si-hsa_circ_0067842-2. B CCK-8 assays in hsa_circ_0067842-overexpressing MCF-7 or hsa_circ_0067842-depleted MDA-MB-231 cells. C Colony formation assays in hsa_circ_0067842-overexpressing MCF-7 or hsa_circ_0067842-depleted MDA-MB-231 cells; Figure S3. The relationship among hsa_circ_0067842, HuR, and CMTM6. A The binding sites of hsa_circ_0067842 and HuR predicted by RBP suite. B Secondary structure of hsa_circ_0067842 and high probable binding regions. C The transfection efficiency of si-CMTM6s in BT-549 and MCF-7 cells was verified by qRT-PCR and western blot. D The expression of CMTM6 in rescue experiments was assessed by qRT-PCR and western blot [file 13062_2023_397_MOESM1_ESM.docx]

**Additional file 1: supplementary figures and figure legends**


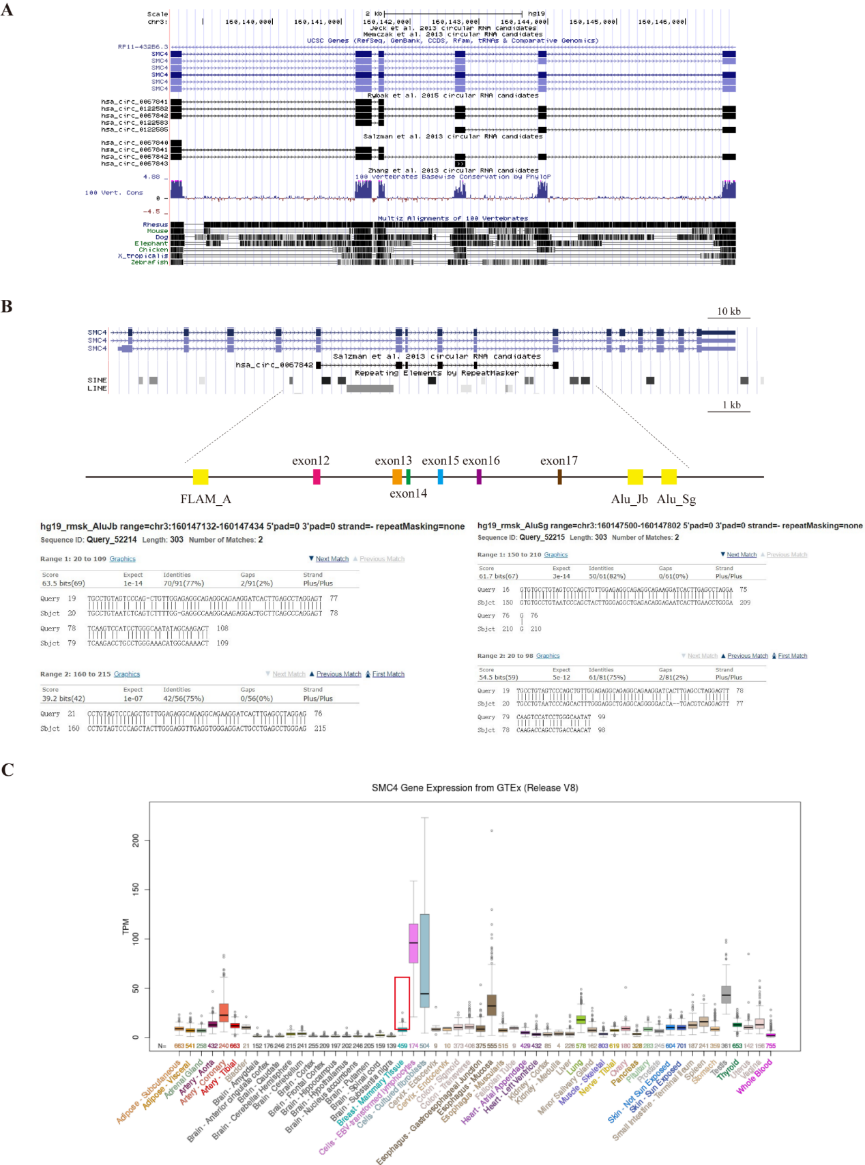


**Figure S1.** **The genomic locus and characteristics of hsa_circ_0067842**

**A** Schematic diagram showing the conservation of hsa_circ_0067842. **B** Schematic diagram showing the genomic locus and the flanking introns with ALU sequence of hsa_circ_0067842 (upper). Complementary ALU sequence (FLAM_A-AluJb and FLAM_A-AluSg) in the flanking introns of SMC4 exon 12 to exon 17 (lower). **C** Expression of SMC4 across diverse normal human tissues from GTEx (https://www.gtexportal.org/home/index.html).


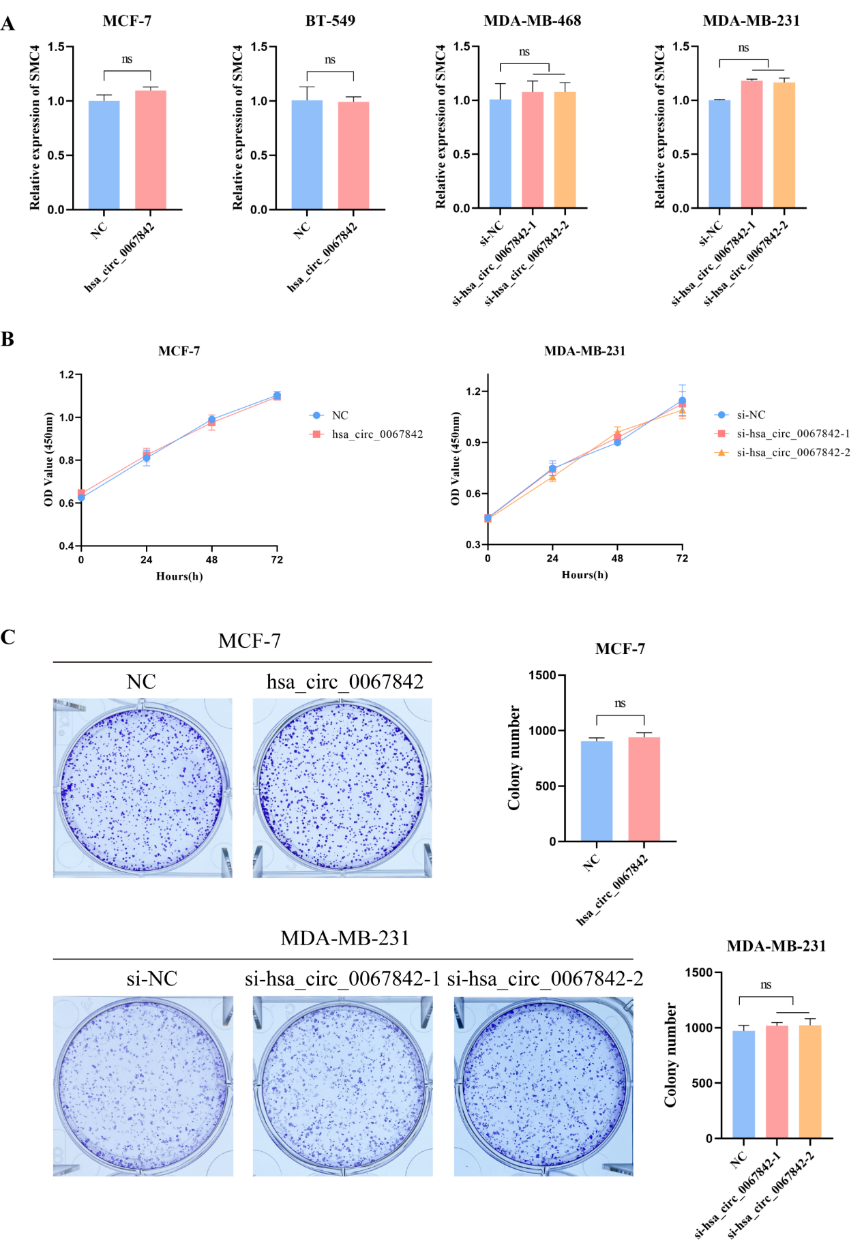


**Figure S2.** **The effects of hsa_circ_0067842 on proliferation of BC cells in vitro.**

**A** qRT-PCR detecting the expression of SMC4 in BC cells after transfection of the NC or hsa_circ_0067842 plasmid and siRNAs si-NC, si-hsa_circ_0067842-1, si-hsa_circ_0067842-2. **B** CCK-8 assays in hsa_circ_0067842-overexpressing MCF-7 or hsa_circ_0067842-depleted MDA-MB-231 cells. **C** Colony formation assays in hsa_circ_0067842-overexpressing MCF-7 or hsa_circ_0067842-depleted MDA-MB-231 cells.


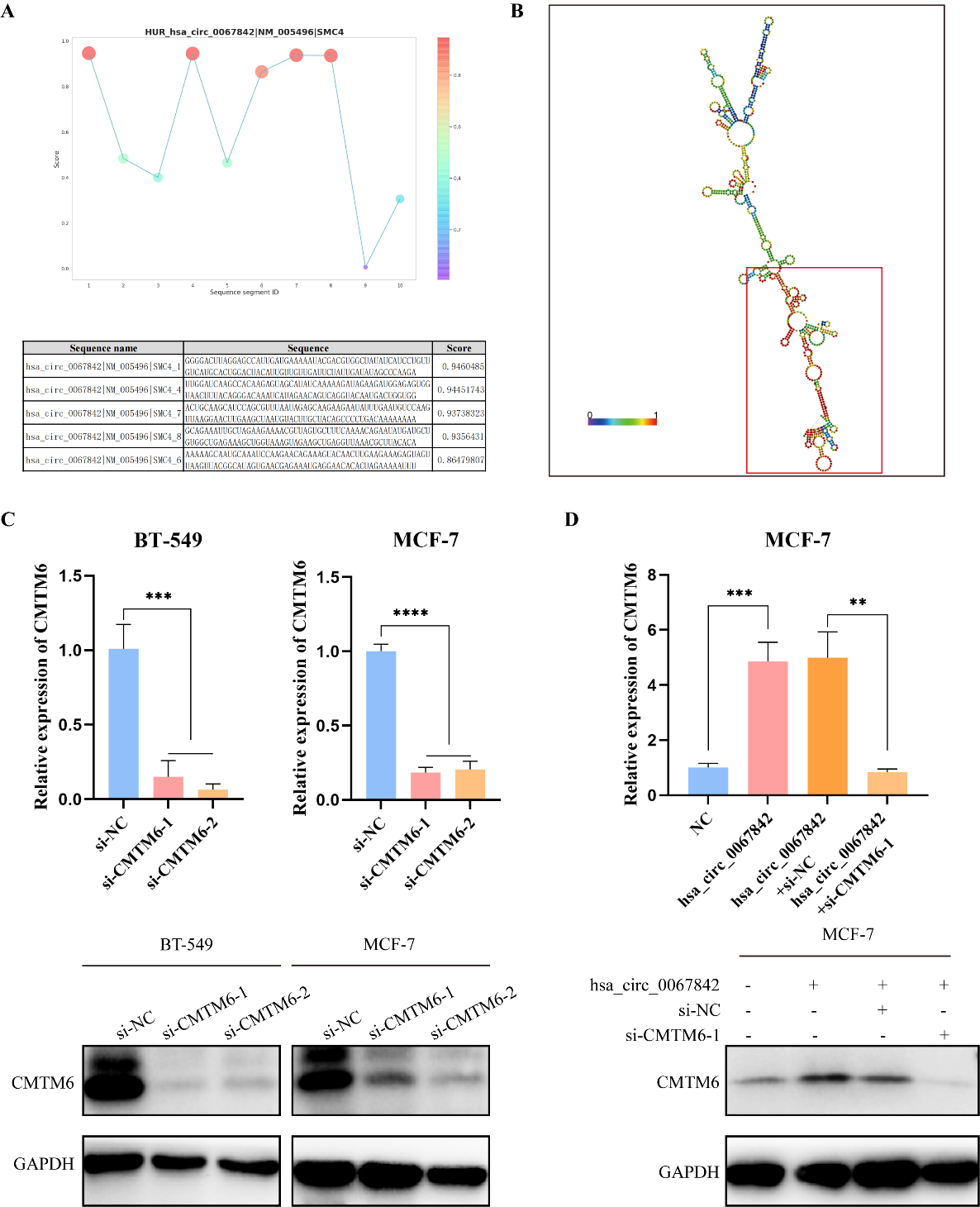


**Figure S3.** **The relationship among hsa_circ_0067842, HuR, and CMTM6.**

**A** The binding sites of hsa_circ_0067842 and HuR predicted by RBP suite. **B** Secondary structure of hsa_circ_0067842 and high probable binding regions. **C** The transfection efficiency of si-CMTM6s in BT-549 and MCF-7 cells was verified by qRT-PCR and western blot. **D** The expression of CMTM6 in rescue experiments was assessed by qRT-PCR and western blot.
