# Supplementary material for: Circular RNA hsa_circ_0067842 facilitates tumor metastasis and immune escape in breast cancer through HuR/CMTM6/PD-L1 axis
Source: Biol Direct. 2023 Aug 18;18:48. doi: 10.1186/s13062-023-00397-3 (PMC10436663; doi:10.1186/s13062-023-00397-3)
Supplement: Supplementary file 2 — Additional file 2: Table S1. Correlation between hsa_circ_0067842 expression and clinicopathological characteristics; Table S2. Sequences of primers used for qRT-PCR in this study; Table S3. Sequences of probes used in this study; Table S4. Antibodies for Immunoblotting in this study; Table S5. Oligonucleotides used in the study [file 13062_2023_397_MOESM2_ESM.docx]

**Additional file 2: supplementary tables**

**Table S1. Correlation between hsa_circ_0067842 expression and** **clinicopathological characteristics**

|  | variables | hsa_circ_0067842 expression | | total | χ^2^ | p value |
| --- | --- | --- | --- | --- | --- | --- |
|  |  | low | high |  |  |  |
| Age (year) |  |  |  |  | 3.774 | 0.052 |
|  | ≤51 | 44 | 6 | 50 |  |  |
|  | >51 | 56 | 20 | 76 |  |  |
| Grade |  |  |  |  | 0.361 | 0.548 |
|  | II | 71 | 20 | 91 |  |  |
|  | III | 29 | 6 | 35 |  |  |
| T stage |  |  |  |  | 2.259 | 0.133 |
|  | T1 | 34 | 13 | 47 |  |  |
|  | T2/T3 | 66 | 13 | 79 |  |  |
| N stage |  |  |  |  | 1.130 | 0.288 |
|  | N0 | 54 | 11 | 65 |  |  |
|  | N1-N3 | 46 | 15 | 61 |  |  |
| TNM stage |  |  |  |  | 0.787 | 0.375 |
|  | I/II | 67 | 15 | 82 |  |  |
|  | III | 33 | 11 | 44 |  |  |
| PR |  |  |  |  | 0.553 | 0.457 |
|  | Negative | 49 | 11 | 60 |  |  |
|  | Positive | 48 | 15 | 63 |  |  |
| E-cad |  |  |  |  | 0.284 | 0.594 |
|  | Negative | 30 | 10 | 40 |  |  |
|  | Positive | 54 | 14 | 68 |  |  |
| HCK |  |  |  |  | 0.303 | 0.582 |
|  | Negative | 38 | 8 | 46 |  |  |
|  | Positive | 13 | 4 | 17 |  |  |
| SMA |  |  |  |  | 0.420 | 0.517 |
|  | Negative | 26 | 7 | 33 |  |  |
|  | Positive | 13 | 2 | 15 |  |  |
| S-100 |  |  |  |  | 0.241 | 0.624 |
|  | Negative | 12 | 3 | 15 |  |  |
|  | Positive | 22 | 8 | 30 |  |  |
| P53 |  |  |  |  | 0.736 | 0.391 |
|  | Negative | 45 | 15 | 60 |  |  |
|  | Positive | 12 | 2 | 14 |  |  |
| Bcl-2 |  |  |  |  | 0.215 | 0.643 |
|  | Negative | 18 | 6 | 24 |  |  |
|  | Positive | 13 | 3 | 16 |  |  |

**Table S2.** **Sequences of primers used for qRT-PCR in this study**

| Item | Sequence | |
| --- | --- | --- |
| hsa_circ_0067842 (divergent) | Forward (5’-3’) | CAAGGCCCAACAAGACAAACTTGA |
|  | Reverse (5’-3’) | ATGGCTCCTAAGTCCCCCTG |
| SMC4 (convergent) | Forward (5’-3’) | GGCAGTGGCAAATCCAATGTT |
|  | Reverse (5’-3’) | ACAACTCTGAATGTCCTTGTGTT |
| GAPDH (divergent) | Forward (5’-3’) | GTATTGGGCGCCTGGTCACC |
|  | Reverse (5’-3’) | CGGCTGGCGACGCAAAAGAA |
| GAPDH (convergent) | Forward (5’-3’) | GCACCGTCAAGGCTGAGAAC |
|  | Reverse (5’-3’) | TGGTGAAGACGCCAGTGGA |
| β-actin | Forward (5’-3’) | CATGTACGTTGCTATCCAGGC |
|  | Reverse (5’-3’) | CTCCTTAATGTCACGCACGAT |
| CMTM6 | Forward (5’-3’) | AGGATGTGTGTTTTTGTTGGCA |
|  | Reverse (5’-3’) | TGGGACTCCTGTCGTTTTTCA |

**Table S3.** **Sequences of probes used in this study**

| Item | Sequence |
| --- | --- |
| hsa_circ_0067842 FISH probe | 5’Cy3-CCTAAGTCCCCCTGTCAGCAGTCTT-3’Cy3 |
| 18S FISH probe | 5’Cy3-CTGCCTTCCTTGGATGTGGTAGCCGTTTC- 3’Cy3 |
| hsa_circ_0067842 pull-down sense probe | Biotin-AATGGCTCCTAAGTCCCCCTGTCAGCAGTCTTGA |
| hsa_circ_0067842 pull-down antisense probe | Biotin-TCAAGACTGCTGACAGGGGGACTTAGGAGCCATT |

**Table S4. Antibodies for Immunoblotting in this study**

| Protein Name | Company | Catalog Number |
| --- | --- | --- |
| HuR | Abcam | ab200342 |
| CMTM6 | Altas | HPA026980 |
| PD-L1 | Cell Signaling Technology | 13684T |
| Ki-67 | Abcam | ab15580 |
| APC anti-human CD3 | Biolegend | 300411 |
| FITC anti-human CD8 | Biolegend | 344703 |
| Ubiquitin | Abcam | ab134953 |
| β-actin | Cell Signaling Technology | 3700S |
| GAPDH | Cell Signaling Technology | 5174S |
| β-Tubulin | Cell Signaling Technology | 2128 |
| Lamin B1 | Proteintech | 12987-1-AP |
| Anti-rabbit IgG, HRP-linked Antibody | ZSGB-BIO | ZB2301 |
| Anti-Rabbit IgG LCS, HRP-linked Antibody | Abbkine | A25022 |
| Anti-rabbit IgG (H+L), F(ab')2 Fragment (Alexa Fluor 555) | Cell Signaling Technology | 4413S |
| Anti-rabbit IgG (H+L), F(ab')2 Fragment (Alexa Fluor 488) | Cell Signaling Technology | 4412S |

**Table S5. Oligonucleotides used in the study**

| Item | Sequence | |
| --- | --- | --- |
| hsa_circ_0067842 siRNA-1 | Sense (5’-3’) | UGCUGACAGGGGGACUUAGTT |
|  | Antisense (5’-3’) | CUAAGUCCCCCUGUCAGCATT |
| hsa_circ_0067842 siRNA-2 | Sense (5’-3’) | GACUGCUGACAGGGGGACUTT |
|  | Antisense (5’-3’) | AGUCCCCCUGUCAGCAGUCTT |
| HuR  siRNA-1 | Sense (5’-3’) | GACGCCAACUUGUACAUCATT |
|  | Antisense (5’-3’) | UGAUGUACAAGUUGGCGUCTT |
| HuR  siRNA-2 | Sense (5’-3’) | GACCAUGACAAACUAUGAATT |
|  | Antisense (5’-3’) | UUCAUAGUUUGUCAUGGUCTT |
| CMTM6  siRNA-1 | Sense (5’-3’) | CUGUGAAGAAGUUGUAUCATT |
|  | Antisense (5’-3’) | UGAUACAACUUCUUCACAGTT |
| CMTM6  siRNA-2 | Sense (5’-3’) | GUCUCCUUAUAACUGAUUGUTT |
|  | Antisense (5’-3’) | ACAAUCAGUAUAAGGAGACTT |
